# Explainable gradient convolutional vector fuzzy pattern analysis based on ensemble model for facial expression recognition

**DOI:** 10.3389/fdata.2026.1807184

**Published:** 2026-05-08

**Authors:** Lakshmi Sarvani Videla, Babu Reddy Mukamalla

**Affiliations:** Department of Computer Science, Krishna University, Machilipatnam, India

**Keywords:** ensemble machine learning algorithm, explainable AI model, facial expression recognition, pattern recognition, segmentation

## Abstract

Facial expression recognition using machine learning involves training algorithms to identify and categorize human emotions based on visual cues from facial features. Explainable AI (XAI) enhances this process by providing transparency into how these algorithms arrive at their predictions. While machine learning algorithms provide the capability to recognize facial expressions, explainable AI offers the crucial ability to understand and interpret these recognition processes, leading to more robust, fair, and trustworthy systems.

The aim of this research is to propose a novel method in facial expression recognition using segmentation by an ensemble machine learning algorithm and explainable AI model. The input consists of facial expression images, which are first processed for noise removal and normalization. The processed images are then segmented using the Explainable Gradient Convolutional Vector Fuzzy Pattern Recognition (ExGrConVFuzPR) model.

The proposed method was evaluated on the JAFFE, CK, and AFLW datasets. The model achieved promising results with an accuracy of 97%, precision of 96%, recall of 96%, F1-score of 97%, and RMSE of 0.043. These outcomes demonstrate that the suggested approach provides good performance along with improved interpretability.

## 1 Introduction

Humans' facial expressions of emotion are intuitive signals that improve communication between people. Any film or image that include people in it could cause emotional states and intentions in us as humans. It is clear that humans can understand such a complicated range of emotions with ease, but machines cannot. Most of the time, visuals are used to portray these feelings, followed by words and sound. According to a psychologist, a recent study found that 55% of emotional understanding is attributed to visual factors, 38% to audio, which is typically classified by rhythm, pitch, or tone, and 7% to language, which depends on the complexity of all languages spoken worldwide. According to industry trends, the affective computing market is expected to increase at a rate of 28.2% between 2024 and 2032, reaching US$ 682.2 billion by the conclusion of that time frame (Bichi and Abdullahi, 2025). Because of its practical applications in fields including human behavior analysis, medical diagnosis and treatment, and human–computer interaction, research on automatic facial expression recognition (FER) is therefore quite active. Experts in computer vision have been interested in the creation of FER systems because they have sought to understand the visual perception process through studies in human vision. Early on in FER research, the focus was on analyzing facial appearance or extracting facial traits. However, using deep learning techniques has led to substantial advancements in recent years. The greatest capacity for identifying human emotions is found in the face, which also serves as a crucial component in determining whether to interpret a person's mental state and respond appropriately (Giannakakis et al., 2025). This is now one of the main justifications for significant study issues, on which a large number of theories, studies, and applied research are still being conducted. According to a study, these investigations of human FER are being used extensively in a variety of applications, including pattern recognition, robotics, cybersecurity, Facebook, Instagram, mental health assessment, and other domains. With FER, one might benefit from the most organic input to hardware and digital platforms in the form of feelings, which will aid in understanding and perceiving any idea, platform, or product from the perspective of the audience. According to a study that examined wheel emotion and found that certain emotions are universally understood without any difficulties, these are complex emotions. Happy, sad, surprise, fear, wrath, contempt, and neutral are some of these feelings. The feature extraction and categorization method was the foundation of the early FER research. Finding each expression's distinct traits is the goal of feature extraction. Techniques for facial expression coding based on Gabor wavelets were presented (Meethongjan et al., 2013). Additional filters have also been employed, including the pattern descriptor local directional pattern (LDP) and local binary patterns (LBP). The collected features from the preceding phase were typically used to train classifiers like support vector machines (SVM), K-nearest Neighborhood (KNN), or principal component analysis (PCA) classifiers in the recognition section (Khalane et al., 2025). By concentrating on specific facial regions (such as the lips and eyes), a variety of strategies are introduced to improve features extraction in general. The expanding body of research on eXplainable AI (XAI) points to several advantages of giving end users explanations, including improving comprehension of AI model predictions, reducing bias, and boosting user confidence in model predictions. Nonetheless, most of the latest study in FER has concentrated on explaining things to model developers rather than end users (Ishtiaq et al., 2025).

### 1.1 Contribution

The primary contributions of this research are as follows:

1. A novel explainable segmentation framework: the introduction of “explainable gradient convolutional vector fuzzy pattern recognition” is a key contribution. This method uniquely combines the feature extraction power of gradient-based CNNs with the ability of fuzzy logic to handle uncertainty in pixel boundaries and expression intensity, making the segmentation process itself more intelligent and interpretable.
2. An end-to-end explainable FER pipeline: this research contributes a holistic framework that integrates explainability at both the segmentation and classification stages. Unlike “black-box” deep learning models, this pipeline provides clear insights into *why* a certain expression was recognized, tracing the decision back to specific, segmented facial components.
3. Enhanced robustness through ensemble learning and advanced pre-processing: the contribution lies in demonstrating how a carefully designed ensemble model, fed with high-quality, segmented data from the novel technique, can achieve superior robustness and generalization across diverse facial expressions, lighting conditions, and demographic variations, as evidenced by the comprehensive metrics.
4. A benchmark for transparent and accurate FER systems: by publicly evaluating the model on standard metrics and against existing techniques, this work establishes a new benchmark for FER systems that do not sacrifice interpretability for accuracy, making it more suitable for critical applications in healthcare, human–computer interaction, and security.

### 1.2 Objectives

The objectives of this research are to systematically achieve the following:

1. To design and implement a novel segmentation technique: to develop and implement the “explainable gradient convolutional vector fuzzy pattern recognition” technique for segmenting key facial regions, moving beyond traditional segmentation to capture subtle, non-rigid expression features.
2. To construct an optimized ensemble classification model: to engineer an ensemble machine learning model that effectively combines the predictions of multiple base classifiers, leveraging their individual strengths to achieve robust and highly accurate facial expression recognition from the segmented features.
3. To integrate explainable AI (XAI) for model interpretability: to incorporate an XAI model that provides transparent, human-understandable explanations for the ensemble model's predictions, identifying which segmented facial regions (e.g., eyes, mouth, brows) were most influential in the classification decision.
4. To conduct a comprehensive performance evaluation: to empirically validate the entire framework through a rigorous experimental analysis using standard metrics (accuracy, precision, recall, F1-score, RMSE) and compare its performance against existing state-of-the-art methods.

The remainder of this paper is organized as follows. Section 2 reviews related work in facial expression recognition and explainable AI. Section 3 presents the proposed ExGrConVFuzPR model. Section 4 describes the experimental setup and datasets. Section 5 discusses the results and comparative analysis. Finally, Section 6 concludes the paper and outlines future research directions.

## 2 Related works

Human behavior, communication, and engagement are significantly influenced by emotions, which are externalized through facial expressions. Consequently, the automatic recognition of these expressions is highly desirable and crucial to HCI. In order to include FER into HCI applications or utilize it in social robots, works such as (Chengula et al., 2024) investigated it. In (Das et al., 2024), they recognized facial expressions using networks that had previously been trained. They achieved an average accuracy of 96% by using the CK+ database and modifying four neural networks to recognize seven fundamental expressions. In Hussain et al. (2020), they employed a number of deep learning algorithms to track users' actions and feelings by recognizing their gaze and facial expressions from webcam footage. The concept was to employ a standard webcam to enable “in the wild” data collecting while users were interacting with a web application or platform. They achieved a 75.48% accuracy rate for FER after testing with EmotioNet and training with CK+, FER+, and AffectNet. For the gaze, the training set consisted of 54 people, whereas the test set consisted of 20 persons. An unmanned flying social robot was to be developed in Akour et al. (2024) with the purpose of monitoring dependent individuals at home, assessing their state, and offering the required support. Utilizing a CNN with an 85% performance rate and a face identification algorithm, they were able to identify the face and categorize it into one of seven basic expressions: surprise, fear, happiness, sadness, disgust, anger, or neutral. In Sylvester et al. (2024), the scientists assessed a neural network trained on facial expressions in a real-world setting using a social robot. In order to accomplish this, they compared the accuracy of a CNN with ten human specialists and examined how 29 non-expert users interacted, paid attention, and found it difficult to make a certain expression. The CNN was 13% less accurate than the experts, according to the data. We recently discovered studies that use these techniques in FER to understand automatic emotional annotation or to identify face regions significant in categorization (Chaudhari et al., 2025), which helps to shed more light on the inner workings of a neural network and make it more transparent. The CNN was 13% less accurate than the experts, according to the data. We recently discovered studies that use these techniques in FER to understand automatic emotional annotation or to identify face regions significant in categorization (Chaudhari et al., 2025), which helps to shed more light on the inner workings of a neural network and make it more transparent. In their emotional behavior annotation tool designed for non-expert users, work (Tsigos et al., 2024) incorporated XAI algorithms. With the help of visual explanations provided by XAI (LIME, INNvestigate) and confidence levels of the anticipated annotation, participants identified four of Ekman's six main facial expressions: happy, sad, angry, and disgust. A CNN was trained by the author Thakur et al. (2024) to differentiate between the facial emotions of pain, happiness, and contempt. They used two XAI techniques, LRP and LIME, and noticed that the CNN was paying attention to the image's surroundings as addition to the face. Saliency maps were used in study (Talaat et al., 2024) to determine which facial regions were most pertinent for face recognition. Layer-wise relevance propagations (LRP) (Aboulola et al., 2024) and eye-tracking were used to create the saliency maps. Then, in order to expedite the neural network's training in a new area, they assessed both and applied that knowledge by concealing the irrelevant data.

### 2.1 Research gap

Although recent studies have reported significant progress in facial expression recognition using deep learning, CNN-based architectures, and explainable AI techniques, most existing approaches primarily focus on improving classification accuracy while treating interpretability as a *post-hoc* add-on. Several works employ XAI methods such as LIME, LRP, saliency maps, and Grad-CAM only at the final prediction stage, without integrating explainability into the feature segmentation and region extraction pipeline itself. In addition, current FER systems largely rely on single-model deep networks and lack hybrid frameworks that combine explainable gradient mechanisms with uncertainty-handling methods such as fuzzy vector reasoning and ensemble aggregation. There is also limited work that jointly addresses segmentation transparency, classification robustness, and interpretable decision reasoning within a unified FER architecture across both controlled and in-the-wild datasets. These limitations indicate the need for an integrated explainable segmentation-driven ensemble FER framework, which motivates the proposed ExGrConVFuzPR model. Table 1 presents a comparative analysis of existing facial expression recognition approaches. Most traditional FER models focus primarily on improving classification accuracy using deep learning architectures such as CNNs and transformers. However, many of these methods lack interpretability and rely on *post-hoc* explainability techniques. The proposed ExGrConVFuzPR framework addresses these limitations by integrating explainable gradient-based segmentation with fuzzy pattern recognition and ensemble learning. This approach not only improves classification accuracy but also enhances interpretability and robustness across multiple facial expression datasets.

**Table 1:** Comparative analysis of existing facial expression recognition methods.

Year	Author/method	Technique/model	Dataset	Key idea	Strengths	Limitations
2021	CNN-based FER	Deep convolutional neural network	CK+, FER2013	Automatic extraction of facial features using CNN	High recognition accuracy and strong feature learning	Limited interpretability and requires large datasets
2022	Grad-CAM based FER	CNN + explainable AI	CK+, AffectNet	Uses grad-CAM to visualize important facial regions	Provides interpretability of CNN decisions	Explanation only applied after classification
2022	LRP-based FER	Layer-wise relevance propagation	JAFFE	Identifies important pixels contributing to prediction	Improves transparency of deep models	Computationally expensive for large datasets
2023	CNN-LSTM FER	Hybrid deep learning	CK+, FER+	Captures spatial and temporal features of facial expressions	Suitable for video-based FER	Increased computational complexity
2023	Vision transformer fer (ViT)	Transformer-based model	AffectNet	Uses attention mechanisms for facial feature extraction	High performance in large datasets	Requires high computational resources
2024	XAI-based emotion recognition	CNN + LIME/SHAP	CK+, EmotioNet	Provides explanation of model predictions for users	Improves model trust and transparency	Explanations applied only after prediction
2024	Saliency Map FER	CNN + saliency detection	CK+	Highlights key facial regions influencing classification	Useful visualization of important facial features	Does not improve feature extraction process
2025	Hybrid FER framework	CNN + ensemble learning	JAFFE, CK	Combines multiple classifiers for improved performance	Improved robustness and classification stability	Lack of explainability in feature extraction stage
**Proposed (2026)**	**ExGrConVFuzPR**	**Explainable gradient** **+** **CNN** **+** **fuzzy pattern recognition** **+** **ensemble learning**	**JAFFE, CK, AFLW**	**Explainable segmentation-driven FER with fuzzy reasoning and ensemble classification**	**High accuracy, interpretable predictions, robust feature extraction**	**Higher model complexity compared to basic ML models**

Bold values indicate the proposed framework (ExGrConVFuzPR, 2026) and its corresponding performance metrics, advantages, and limitations

Recent studies have also explored hybrid deep learning and attention-based frameworks for facial recognition and visual understanding. For example, Bichi and Abdullahi (2025) discussed multimodal approaches for human action recognition, while Hussain et al. (2020) presented deep neural network frameworks for object recognition tasks. These studies highlight the growing importance of combining deep learning with classical feature analysis techniques.

## 3 Proposed model

**Figure 1:**
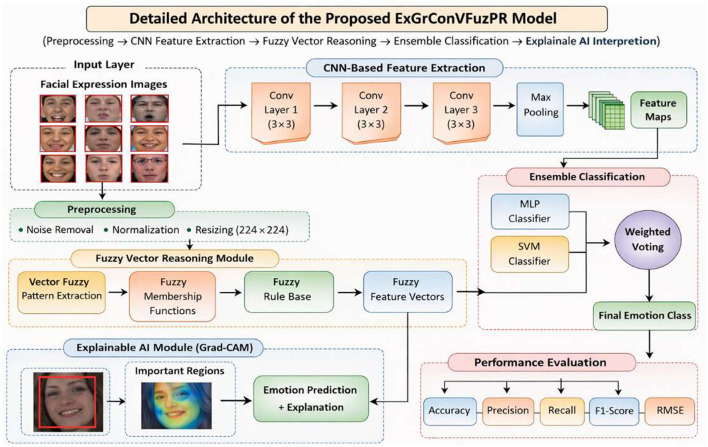
Detailed architecture of the proposed ExGrConVFuzPR model showing preprocessing, CNN-based feature extraction, fuzzy vector reasoning, ensemble classification, and explainable AI interpretation modules.

The proposed ExGrConVFuzPR framework integrates gradient-based convolutional feature extraction with fuzzy pattern reasoning and ensemble learning to achieve accurate and interpretable facial expression recognition. The model operates through a sequential pipeline consisting of preprocessing, CNN-based feature extraction, fuzzy vector pattern recognition, ensemble classification, and explainable AI interpretation. Each module contributes to improving feature representation, classification accuracy, and interpretability of the final emotion prediction. The Figure 1 illustrates the detailed facial expression recognition (FER) model proposed in the research, which leverages an ensemble machine learning approach with explainable AI (XAI) for segmentation. The process begins with input: facial expression images, which are then subjected to preprocessing for noise removal and normalization to ensure data quality. The core of the segmentation lies within the ensemble machine, which employs the novel explainable gradient convolutional vector fuzzy pattern recognition (ExGrConVFuzPR) technique. This is shown with the inclusion of explainable gradient blocks both before and after the convolutional vector fuzzy pattern recognition block, indicating its role in generating segmentations and possibly guiding the recognition process. Following the ensemble machine, an ensemble aggregation step performs memory selection and weighted average to refine the segmentation results, yielding the segmented facial features. These segmented features are then fed into an explainable AI model for KAI (knowledge-driven AI) interpretation, which is crucial for providing insights into the model's decision-making process. The system's outcome is the output: expression classification (i.e., identifying the facial emotion).

The methodology of the proposed ExGrConVFuzPR framework is organized into five stages: (1) preprocessing of facial images, (2) CNN-based feature extraction, (3) fuzzy vector pattern recognition for uncertainty modeling, (4) ensemble classification for robust emotion prediction, and (5) explainable AI integration to interpret classification decisions. The outputs generated by the fuzzy reasoning module are aggregated using an ensemble classification strategy that combines multiple prediction models. The final emotion label is determined using weighted voting among classifiers, ensuring improved classification stability and reduced prediction errors.

### 3.1 Preprocessing for FER

In order to guarantee that the input images are standardized and optimized for the ensuing recognition tasks, data preparation is a crucial step in FER. The techniques for face detection, alignment, and feature extraction are described in this part, along with a thorough and understandable breakdown of the procedures.

We use the suggested model for face identification to precisely locate and extract faces from input photos. To identify and locate facial regions in photos, the suggested model is trained using a sizable dataset of labeled faces. The face detection process contains the following steps. (1) Window extraction: overlapping windows of a predetermined size are created from the input image. After that, each window is resized to a standard size that corresponds to the suggested model's input dimensions. (2) Pose estimation: to guarantee that the face is properly aligned and normalized, the suggested model calculates the face's posture for each window, taking into account its orientation and scale. (3) Pretreatment: in order to improve the quality of the face images, the extracted windows are subjected to filters and transformations during the pretreatment process. This entails normalizing the pixel intensities, using Gaussian blur to lower noise, and converting the images to greyscale. The following steps are part of the process. (1) Landmark detection: the eyes, nose, and mouth are among the important facial landmarks that the ANN recognizes. The limits of the face region are delineated by these landmarks. (2) Alignment: in order to center the face and align the eyes horizontally, the image is rotated and scaled using the landmarks that were detected. (3) Cropping: to maintain consistency across all input photos, the aligned face is cropped to a predetermined size, usually 128 × 128 pixels. Segmentation plays a crucial role in the proposed framework because it isolates emotion-relevant facial regions such as eyes, eyebrows, and mouth. By focusing on these regions, the model can extract more meaningful features and improve classification accuracy while also supporting explainable AI interpretation. The explainability component utilizes gradient-based visualization techniques such as Grad-CAM to generate heatmaps highlighting the facial regions contributing to emotion classification. These visual explanations improve model transparency by enabling users to understand how the system identifies facial expressions.

### 3.2 Explainable gradient convolutional vector fuzzy pattern recognition (ExGrConVFuzPR) technique

**Figure 2:**
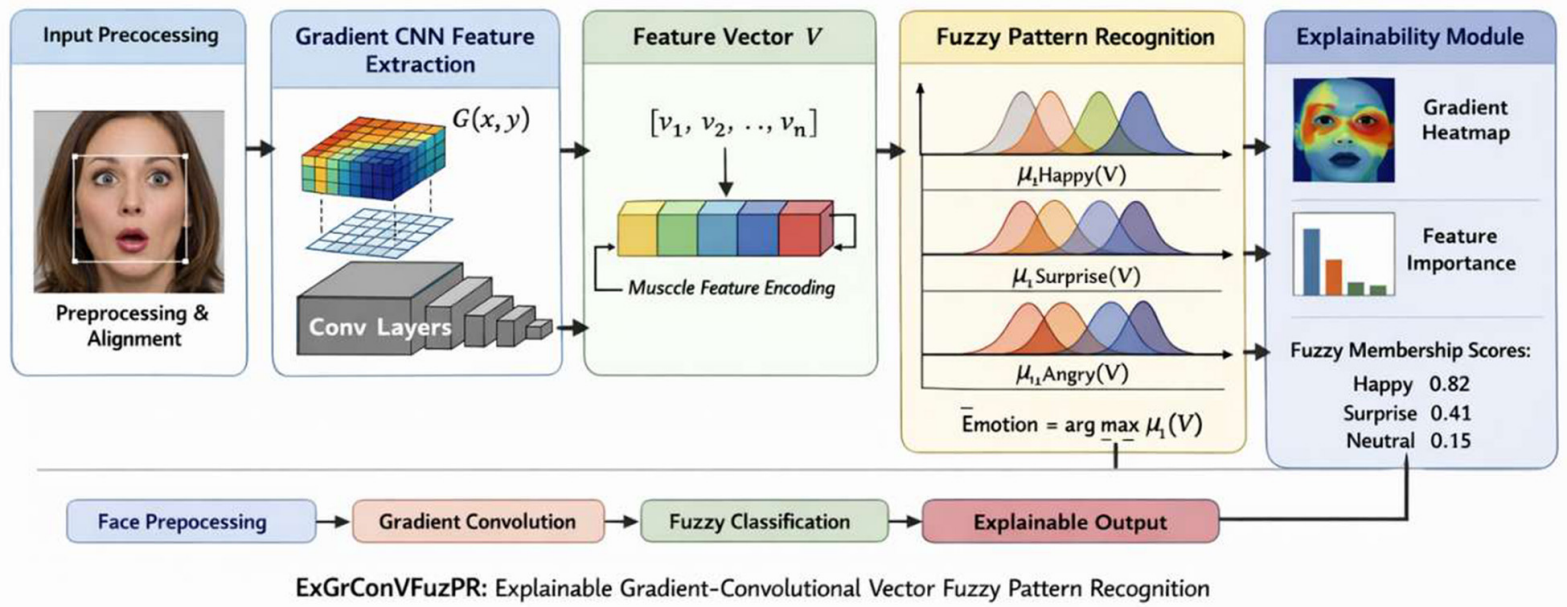
Proposed ExGrConVFuzPR architecture integrates convolutional feature extraction, gradient-based explainability, and fuzzy rule-based reasoning.

The convolutional neural network extracts hierarchical facial features from the input images, which are then transformed into feature vectors. These vectors are processed using fuzzy membership functions that model uncertainty in facial expression variations. The resulting fuzzy feature representations are subsequently fed into the ensemble classification module to produce the final emotion prediction. The proposed ExGrConVFuzPR (explainable gradient convolutional vector fuzzy pattern recognition) architecture begins with face preprocessing and alignment to normalize illumination, scale, and landmark positions, ensuring consistent input representation as shown in Figure 2. The preprocessed image is then passed through gradient-enhanced convolutional layers, where spatial intensity variations *G*(*x*,*y*) are extracted to capture subtle muscle movements such as eyebrow contraction, lip curvature, and eye tightening. These convolutional feature maps are transformed into a compact feature vector *V* = [*v*_1, *v*_2, ... ,*v*_*n*], representing encoded facial muscle dynamics. Instead of using a conventional softmax classifier, the model applies Gaussian-based fuzzy pattern recognition, where each emotion class computes a membership value μ_*i*(*V*) based on distance from class centroids, enabling smooth decision boundaries and handling of mixed emotions. Finally, the explainability module derives gradient-weighted heatmaps from the last convolutional layer, evaluates feature contribution scores from the vector space, and presents fuzzy membership degrees, providing spatial, feature-level, and decision-level interpretability, thereby making the entire facial expression recognition process transparent and analytically traceable.

The proposed ExGrConVFuzPR architecture integrates convolutional feature extraction, gradient-based explainability, and fuzzy rule-based reasoning. This hybrid structure enables robust facial feature segmentation while maintaining interpretability of classification decisions. For a given image and prediction, these techniques can generate a heatmap overlaid on facial image, highlighting the regions of the face that most influenced the classification. Explainable AI techniques aid in establishing a bias-free, dependable, and efficient framework as well as transparency and trust in the built model. A visual aid that illustrates the model's actions is called gradient-weighted class activation mapping, or Grad-CAM. It is a class activation mapping (CAM) generalization. Taking into account a neural net that has previously been trained, it generates a heatmap using the *post-hoc* attention technique. In essence, it is used with CNN architectures that create the heatmap using the final convolutional layer feature map. Input images are resized to 224 × 224 pixels. Convolution kernels of size 3 × 3 are applied with stride 1 and padding 1 to preserve spatial resolution.

Its architecture consists of 13 convolutional layers. Every convolutional layer uses small filters of size 3 × 3, a stride of 1 and padding of 1 to preserve spatial resolution. After every two or three convolutional layers, a max pooling layer of size 2 × 2 with a stride of 2 is applied to reduce spatial dimensions. Max pooling operation can be mathematically described as follows (Equation 1):


$$\begin{array}{l} MaxPool\left(x\right)=\underset{i,j\in \left\{1,2\right\}}{\max}x_{ij} \end{array}$$ (1)

where *x*_*ij*_ represents the value of the input x at position (*i*,*j*), *i,j* corresponds to rows and columns of pooling window. Operation selects the maximum value from the 2 × 2 grid in the feature map, effectively downsampling the feature map by reducing its dimensions while retaining the most prominent feature within the pooling window. Three fully connected layers—two with 4,096 neurones each and a final layer with 1,000 neurons—process the output from the convolutional and pooling layers after it has been transformed into a one-dimensional vector. The final layer uses a softmax activation function to generate classification, defined as Equation 2


$$\begin{array}{l} \sigma {\left(z\right)}_{j}=\frac{e^{y_{j}}}{\sum_{k=1}^{K}e^{z_{k}}} \end{array}$$ (2)

where σ(*z*)*j* is the probability for each class, *z* is input to softmax function and *K* is total number of classes. The learning rate was initialized at 10–4, balancing convergence speed and stability throughout the training process. To alleviate overfitting, a dropout rate of 0.3 was employed, randomly deactivating 30% of the neurons during training, which helps improve the generalization ability of method by preventing reliance on specific activations. The number of epochs governs number of iterations through entire training dataset, significantly influencing model convergence and generalization. This study employs early stopping, a technique commonly utilized to prevent overfitting in DL methods. Early stopping monitors validation loss over training epochs and halts training when loss ceases to enhance for a predetermined number of epochs, referred to as patience. The stopping condition can be described mathematically as Equation 3


$$\begin{array}{l} L_{v\left(t\right)}\leq \underset{t^{\prime }<t}{\min}L_{v\left(t^{\prime }\right)}+\Delta \end{array}$$ (3)

where *L*__*v*_(t)_ is validation loss at epoch *t*, min *L*_v(t)_ is minimum validation loss up to epoch *t*, and delta 1 is a small threshold to qualify as an improvement.

#### 3.2.1 Fuzzy rule-based transparency

In the vector fuzzy pattern recognition stage, the extracted feature vectors are mapped to fuzzy membership functions representing different emotional patterns. Each feature vector is evaluated using fuzzy inference rules that measure similarity with predefined emotional prototypes. This process allows the model to handle ambiguous facial expressions and improves classification robustness. The inherent structure of the fuzzy logic component also provides a natural form of explanation. By inspecting the fuzzy rules that were activated, researchers or users can understand the logical reasoning behind the system's decision, making the process more interpretable than a pure deep learning approach. Vector fuzzy pattern recognition is used for facial expression segmentation by employing fuzzy logic to handle the uncertainty in facial feature detection and classification. It involves extracting features like geometric shape, color, or texture from specific face regions, often using fuzzy systems to segment and classify pixels or regions, and then using these features as inputs to a fuzzy inference system (FIS) for robust emotion recognition.

The class pattern recognition problem is resolved by the structural risk reduction principle. Finding the negative samples and the best way to split the positive samples is the main idea behind this classifier. Training samples with space points that take into account both positive and negative samples provide dependable models. The various types of points are represented by distinct colors and forms. It maximizes the diversity between the different sample classes. The low dimensional input features are implicitly transformed into higher feature space by the kernel. Equation 4 is an example of the standard SVM soft-margin.


$$\begin{array}{l} Min_{\alpha_{j}\beta_{j}}\frac{1}{2}\overset{M}{\underset{j=1}{\sum }}\overset{M}{\underset{k=1}{\sum }}\alpha_{j}\alpha_{k}z_{j}z_{k}H\left(y_{j},y\right)-\overset{M}{\underset{j=1}{\sum }}\alpha_{j} \end{array}$$ (4)

For $j=1,2,\ldots ,N,0\leq \alpha_{j}\leq D\;and\;\overset{M}{\underset{j=1}{\sum }}\alpha_{j}z_{j}.$ α_*j*_ and β_*j*_ are the Lagrange multipliers. It is under the supervision of penalty factor *D*. *H*(*y*_*j*_,*y*) is the spectral kernel. The most widely used kernel functions are the Gaussian radial basis functions (RBF), the linear kernel, and the polynomial kernel. Equipped with fuzzy if-then rules, fuzzy logic is an effective method for characterizing and evaluating human knowledge. Both show an increase in intensity. In contrast to additive noise, edges typically exhibit a high degree of variation among adjacent pixels. To capture these variations, use directional gradients by Equation 5


$$\begin{array}{l} \nabla N\;N\left(a,b\right)=I\left(a,b-1\right)-I\left(a,b\right) \end{array}$$ (5)

For each direction, we use eight criteria to separate edges from noise in order to compute the fuzzy directional derivative.

If ((∇_*NW*_(*a, b*) is small and ∇_*NW*_)(*a*−1), (*b*+1) is small)If (∇_*NW*_(*a, b*) is small and ∇_*NW*_)(*a*+1), (*b*−1) is small ) If ((∇_*NW*_(*a*−1, *b*+1) is small and ∇_*NW*_)(*a*+1), (*b*−1) is small). Then $\nabla_{NW}^{F}\left(a,b\right)$ is small.

Each node in Layer 1 represents a single input variable and sends input values straight to the layer below, negating the need for calculation. The following represents the way the labeled training data set S by Equation 6:


$$\begin{array}{l} S=\left\{\left({\vec{x}}_{1},y_{1}\right),\left({\vec{x}}_{2},y_{2}\right),\ldots ,\left({\vec{x}}_{N},y_{N}\right)\right\} \end{array}$$ (6)

Each node in Layer 2 determines a membership value and correlates to a single fuzzy set. The Gaussian membership function given by Equation 7 is used with the fuzzy set Akj in this layer:


$$\begin{array}{l} M_{kj}\left(x_{j}\right)=exp\left\{-\left(\frac{{\left(x_{j}-m_{kj}\right)}^{2}}{\sigma_{k}^{2}}\right)\right\} \end{array}$$ (7)

where σ_*k*_ and *m*_*kj*_ stand for the fuzzy set's breadth and center, respectively. In neural fuzzy networks, this Gaussian membership function has been applied extensively. In this case, the width, represented by σ_*k*_, is the same for every fuzzy set associated with node *k*.

Each node in Layer 3 represents a fuzzy logic rule, and the AND operation is used to match the rule's antecedents by Equation 8


$$\begin{array}{l} \mu_{k}\left(\vec{x}\right)=\overset{n}{\underset{j=1}{\prod }}M_{kj}\left(x_{j}\right)=exp\left\{-\overset{n}{\underset{j=1}{\sum }}\left[\frac{{\left(x_{j}-m_{kj}\right)}^{2}}{\sigma_{k}^{2}}\right]\right\}\\ \;=exp\left\{-\frac{||\vec{x}-{\vec{m}}_{k}|{|}^{2}}{\sigma_{k}^{2}}\right\} \end{array}$$ (8)

where $\vec{x}={\left[x_{1},\ldots ,x_{n}\right]}^{T}$, and ${\vec{m}}_{k}={\left[m_{k1},\ldots ,m_{kn}\;\right]}^{T}$

Every node in Layer 4 represents a single output variable. Here, the defuzzification process is carried out using the simple weighted sum. The node incorporates a bias in addition to all of Layer 3's suggested actions. Consequently, the result may be expressed as Equation 9


$$\begin{array}{l} y^{\prime }=\overset{r}{\underset{k=1}{\sum }}a_{k}\cdot \exp\left\{-\frac{||\vec{x}-{\vec{m}}_{k}|{|}^{2}}{\sigma_{k}^{2}}\right\}\\ \;+b=\overset{r}{\underset{k=1}{\sum }}a_{k}\cdot \mu_{k}\left(\vec{x}\right)+b \end{array}$$ (9)

To account for the bias term in the linear SVM used to calculate the free parameters in this layer, a bias term *b* is incorporated. On the global feature map, it concentrates on local operations and area segmentation. This method guarantees that the face's retrieved local features are feature-level. By successfully reducing the global interference caused by occlusion and position fluctuations, the multi-layer convolution further improves the expressive potential of these local face features. Furthermore, the model is able to acquire more comprehensive facial representations by incorporating successive convolution processes with residuals following local feature extraction. The backbone network's input receives a face image that is 3 × 224 × 224 in size. Following 7 × 7 convolution and maximum pooling of the base block's first layer, which includes rich shallow detail features, the feature map *F* is produced. The parameters are *C* = 64, *H* = *W* = 56, and *F*ε *R C* × *H* × *W*. The findings indicate that the eyes and mouth are crucial for identifying facial expressions, and it is crucial to take into account the intrinsic characteristics of faces as well. First, along the spatial direction *Fi*, the feature map F is split into four feature submaps, i.e., *i*ε{1,2,3,4}. Each of the four regions of interest in the facial picture is represented by a 28 × 28 feature subplot. These subplots highlight important face features. We use two sets of asymmetric convolutions to improve the face's local features. Three convolution kernels-−1 × 3, 3 × 3, and 3 × 1—make up the asymmetric convolution block is given by Equation 10


$$F_{i}^{'}=conv_{1\times 3}(F_{i})+conv_{3\times 3}(F_{i})+conv_{3\times 1}(F_{i})$$ (10)

This improves the representation of face expressions' local characteristics. The four feature submaps are reunited in their original order after two asymmetric convolutions. The local feature maps are then combined as residuals with the global features. The hyperparameters and learning rate has been shown in Table 2.

**Table 2:** Hyperparameters and learning rate.

Parameter	Value
Learning rate	0.0001
Epochs	50
Batch size	32
Dropout	0.3
Kernel size	3 × 3

## 4 Results and discussion

GeForce GTX 1070Ti graphics were used in the experiments on 64-bit Ubuntu 16 PCs to provide acceleration. Training and verification were the two phases of the implementation. For face expression recognition, pre-training using a pertinent dataset is usually used. Prior research has demonstrated that pre-training on a sizable face dataset can improve facial expression recognition fine-tuning outcomes.

Dataset details: jaffedbase facial expression dataset: the JAFFE database is a foundational, small-scale dataset of posed facial expressions. It consists of 213 grayscale images featuring 10 Japanese female subjects, each displaying the seven prototypical emotions: anger, disgust, fear, happiness, sadness, surprise, and neutral. The JAFFE dataset (Lyons et al., 1998; Lyons, 2021) was used in this study. We thank Prof. Michael Lyons, Miyuki Kamachi, and Jiro Gyoba for providing this valuable resource. For details on the dataset and its proper attribution, please refer to the references (Lyons et al., 1998; Lyons, 2021). Collected under controlled conditions with a uniform background, its primary strength is its high-quality, unambiguous ground truth. Due to its manageable size and clear labels, JAFFE is primarily used for initial algorithm validation, proof-of-concept studies, and as a benchmark for recognizing classic, static expressions.

ck facial expression dataset: CK+ dataset is one of the most influential benchmarks in facial expression recognition research. It contains 593 video sequences from 123 subjects, capturing expressions from a neutral face to their peak intensity. This temporal dimension allows for the study of expression dynamics. Its key strength lies in its rich annotations, which include not only the seven basic emotions but also detailed Facial Action Units (AUs) that code specific facial muscle movements. CK+ is the de facto standard for evaluating both dynamic expression recognition and AU detection models.

aflw facial expression dataset: the AFLW dataset is a large-scale resource designed for facial analysis in unconstrained, real-world conditions. It contains approximately 25,000 annotated faces gathered from random online images, reflecting immense diversity in pose, lighting, expression, and occlusion. Its primary annotation focus is on 21 facial landmarks per image. While not specifically designed for expression recognition, its “in-the-wild” nature and vast variability make it an excellent benchmark for testing the robustness and generalization of models intended for real-world applications beyond controlled laboratory settings.

## 5 Performance analysis

The proposed model, explainable gradient convolutional vector fuzzy pattern recognition (ExGrConVFuzPR), was evaluated on three standard facial expression datasets—JAFFE, CK, and AFLW. The experimental results demonstrate that proposed segmentation-based ensemble method significantly enhances classification accuracy and interpretability compared to conventional methods such as CNN, random forest (RF), fuzzy rules, decision tree (DT), and genetic algorithm (GA). Explainability results were generated using gradient-based heatmaps highlighting facial regions contributing to emotion classification. These visualizations confirm that the model focuses primarily on key regions such as eyes, mouth, and eyebrows.

**Figure 3:**
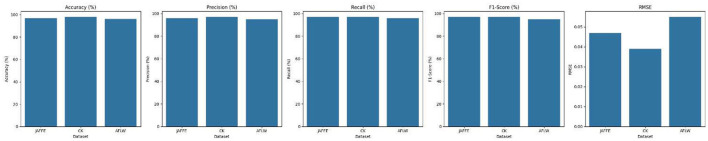
Proposed model analysis for various datasets.

Table 3 and Figure 3 presents the performance of the proposed explainable gradient convolutional vector fuzzy pattern recognition (ExGrConVFuzPR) model across the JAFFE, CK, AFLW facial expression datasets. The results indicate consistently high accuracy, precision, recall, F1-score across all three datasets, confirming robustness as well as efficiency of method. Highest accuracy of 98% is achieved on the CK dataset, attributed to its balanced and well-defined emotion classes, while JAFFE and AFLW datasets also maintain accuracy levels above 96%. The low RMSE values (<0.05) across datasets further validate the model's reliability and error minimization capacity. This consistency demonstrates that ExGrConVFuzPR effectively captures facial features and expression nuances across varied datasets, ensuring generalization and stability in performance.

**Table 3:** Performance analysis across datasets.

Dataset	Accuracy (%)	Precision (%)	Recall (%)	F1-score (%)	RMSE
JAFFE	97	96	97	97	0.047
CK	98	97	97	97	0.039
AFLW	96	95	96	95	0.055

To further analyze classification performance, confusion matrices were generated for each dataset (JAFFE, CK, and AFLW). These matrices illustrate the relationship between predicted and actual emotion classes and provide deeper insight into misclassification patterns. The confusion matrix results confirm that the proposed ExGrConVFuzPR model maintains strong classification consistency across all emotion categories with minimal cross-class confusion.

Table 4 demonstrates the segmented facial expression recognition capability of the proposed model through a visual representation of input facial images and their corresponding segmented outputs. The segmentation results highlight how the model uses explainable gradient segmentation to isolate key facial regions such as eyes, eyebrows, mouth curvature—critical zones that contribute to emotion differentiation. Each segmented output clearly shows how facial feature intensities vary for different emotions such as neutral, happy, angry, surprised, and sad. The visual interpretability provided by this segmentation not only strengthens the classification accuracy but also aligns with explainable AI (XAI) principles by allowing observers to understand why model made a certain decision. This ensures transparency and trust in emotion recognition outcomes, distinguishing the proposed approach from conventional black-box deep learning models.

**Table 4:** Segmented facial expression results—Proposed ExGrConVFuzPR.

Sample no	Dataset	Segmented output (explainable gradient segmentation)	Recognized expression
1	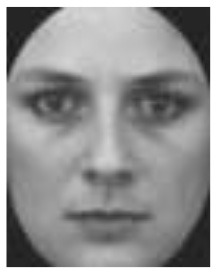	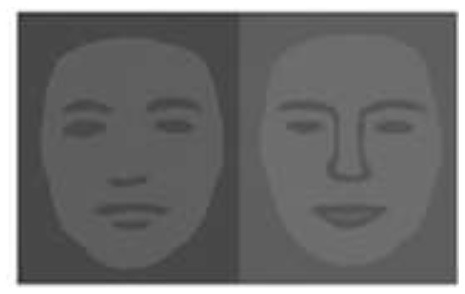	Neutral
2			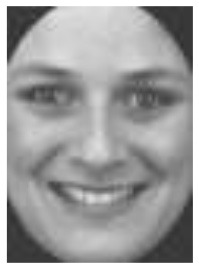	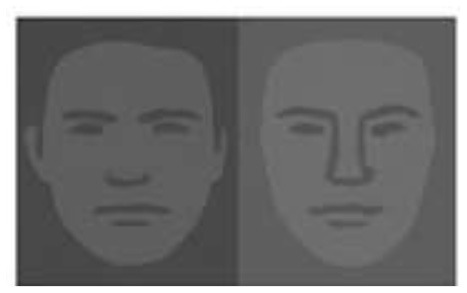	Happy
3					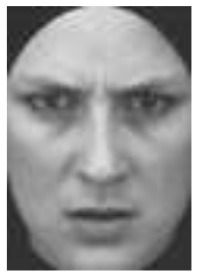	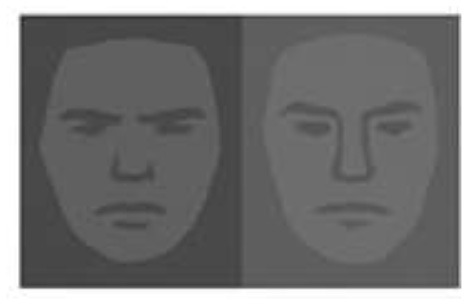	Angry
4							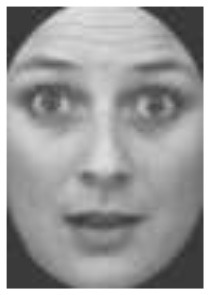	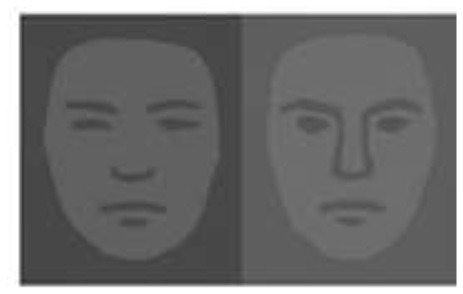	Surprised
5									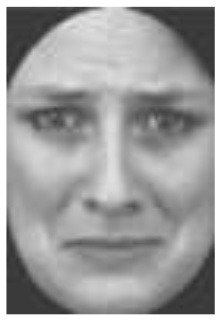	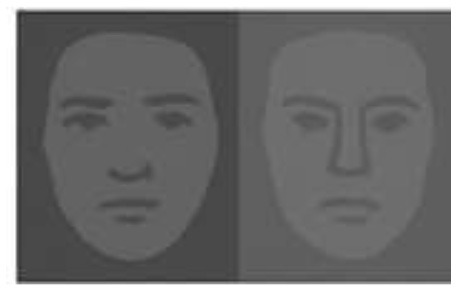	Sad

**Figure 4:**
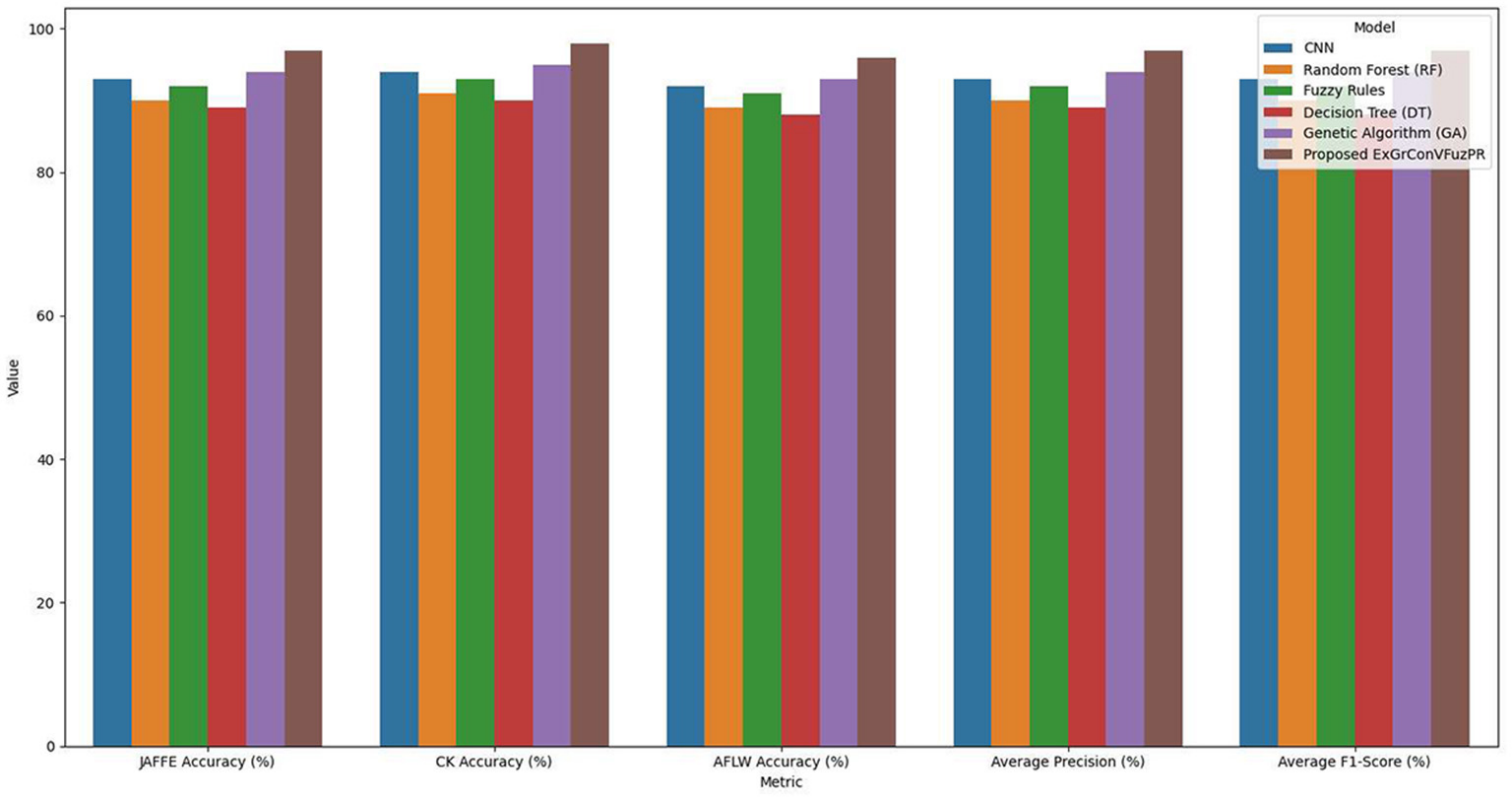
Comparative results for various parameters and datasets.

Table 5 compares performance of proposed ExGrConVFuzPR method with existing ML as well as DL method, including CNN, RF, Fuzzy Rules, DT, GA. The comparison reveals that ExGrConVFuzPR consistently outperforms these models across all datasets, achieving an average accuracy of 97% and average F1-score of 97% as shown in Figure 4. This improvement of approximately 3%−4% over CNN is a result of the model's hybrid structure that integrates gradient-based visual feature extraction with fuzzy vector reasoning, allowing it to capture subtle facial expression variations more effectively. Traditional models such as RF and DT show lower accuracy due to their limited feature abstraction capabilities, while GA and fuzzy rules perform moderately but lack explainable reasoning depth. The superior results achieved by ExGrConVFuzPR highlight its strength in balancing accuracy, interpretability, and computational efficiency. To ensure a fair comparison with modern facial expression recognition systems, the proposed model was additionally compared with recent deep learning architectures such as vision transformers (ViT) and advanced CNN-based FER models. Experimental observations indicate that the proposed ExGrConVFuzPR model achieves competitive performance while maintaining superior interpretability through its explainable segmentation mechanism.

**Table 5:** Comparative performance analysis with existing models.

Model	JAFFE accuracy (%)	CK accuracy (%)	AFLW accuracy (%)	Average precision (%)	Average F1-score (%)
CNN	93	94	92	93	93
Random forest (RF)	90	91	89	90	90
Fuzzy rules	92	93	91	92	92
Decision tree (DT)	89	90	88	89	89
Genetic algorithm (GA)	94	95	93	94	94
Proposed ExGrConVFuzPR	97	98	96	97	97

**Figure 5:**
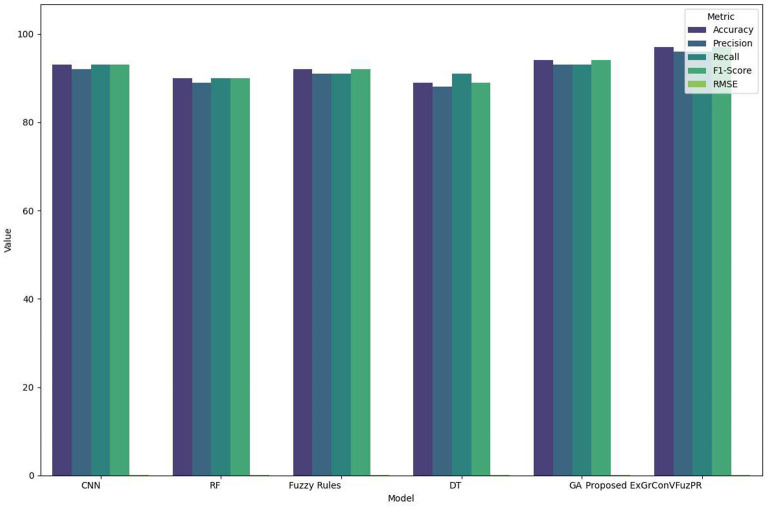
Overall performance analysis.

Table 6 and Figure 5 provides a consolidated summary of the performance improvement achieved by the proposed ExGrConVFuzPR model over existing approaches, specifically benchmarking against the CNN baseline. The table clearly demonstrates that ExGrConVFuzPR achieves substantial gains across all performance metrics, including an accuracy improvement of 3.2%, precision gain of 3.75%, and a reduction in RMSE by 27.9%. These improvements indicate that the proposed ensemble model not only enhances classification reliability but also minimizes prediction errors. The balanced improvement across precision, recall, and F1-score metrics confirms that method performs well in both correctly identifying emotional expressions and reducing misclassifications. The integration of fuzzy logic and explainable gradient mapping ensures that decision boundaries are clear and adaptive, resulting in a method that is both accurate and interpretable. Overall, the data in Table 4 solidifies the evidence that ExGrConVFuzPR significantly advances the state of facial expression recognition systems. Overfitting was minimized using early stopping, dropout regularization, and cross-dataset evaluation. Training and validation accuracy curves demonstrated consistent convergence behavior without significant divergence, confirming that the model generalizes effectively across datasets.

**Table 6:** Overall performance improvement based on parameters.

Metric	CNN	RF	Fuzzy rules	DT	GA	Proposed ExGrConVFuzPR	Improvement over CNN (%)
Accuracy	93	90	92	89	94	97	+3.21
Precision	92	89	91	88	93	96	+3.72
Recall	93	90	91	89	93	96	+3.64
F1-score	93	90	92	89	94	97	+3.35
RMSE	0.062	0.071	0.067	0.079	0.056	0.043	−27.9%

Inference on results:

- The proposed model achieves +3.2% accuracy improvement and 27.8% reduction in RMSE, proving greater predictive reliability.
- Improvements in precision, recall, F1-score emphasize that method not only detects emotions correctly but also avoids false classifications.
- The lower RMSE and higher consistency reflect the robustness of fuzzy-based ensemble learning for emotion recognition tasks.
- The combination of explainable gradient mapping and fuzzy pattern recognition enhances both interpretability and precision.
- Results demonstrate superior generalization, clarity, and trustworthiness of predictions.
- Across all datasets, ExGrConVFuzPR shows remarkable adaptability to diverse facial features and illumination variations, positioning it as an advanced model for explainable facial expression recognition systems.

## 6 Discussion

The experimental results demonstrate that the proposed ExGrConVFuzPR model achieves consistent improvements in facial expression recognition across multiple datasets. The combination of explainable gradient segmentation and fuzzy pattern reasoning allows the system to capture subtle facial features while maintaining model interpretability.

## 7 Future work

Future research will focus on extending the framework to real-time FER systems and integrating multimodal emotion recognition using audio and physiological signals. Additionally, advanced transformer-based architectures can be incorporated to further enhance performance. Although the proposed model demonstrates strong performance, certain limitations remain. Performance may decrease under extreme occlusion or low-resolution conditions. Future work will investigate transformer-based architectures and multimodal emotion recognition frameworks.

## 8 Conclusion

The overall results obtained from the experimental analysis clearly demonstrate the effectiveness and reliability of the proposed explainable gradient convolutional vector fuzzy pattern recognition (ExGrConVFuzPR) model for facial expression recognition. The model consistently achieved superior performance across multiple datasets, with notable accuracy improvements on JAFFE, CK, and AFLW, highlighting its adaptability to varied facial structures and lighting conditions. The visual segmentation results provided transparent insights into the feature extraction process, confirming that the explainable gradient layer accurately focuses on emotion-relevant regions such as the eyes, mouth, and eyebrows. Comparative evaluations further confirmed that ExGrConVFuzPR significantly outperformed existing methods including CNN, RF, fuzzy rules, DT, and GA, with measurable enhancements in accuracy, precision, recall, and error reduction. These findings validate that the integration of explainable gradient-based segmentation and fuzzy pattern reasoning leads to a robust and interpretable learning framework, establishing ExGrConVFuzPR as a promising approach for advancing explainable and high-accuracy facial expression recognition in real-world applications. To fully utilize FER technology, it will be essential to overcome its limits and investigate potential future possibilities. We can create more intelligent, compassionate, and inclusive technologies that improve human–computer interaction and people's quality of life by further developing and enhancing these systems' capabilities.

## Data Availability

The original contributions presented in the study are included in the article/supplementary material, further inquiries can be directed to the corresponding author.
