# Correction: Explainable gradient convolutional vector fuzzy pattern analysis based on ensemble model for facial expression recognition

**DOI:** 10.3389/fdata.2026.1883246

**Published:** 2026-06-10

**Authors:** Lakshmi Sarvani Videla, Babu Reddy Mukamalla

**Affiliations:** Department of Computer Science, Krishna University, Machilipatnam, India

**Keywords:** ensemble machine learning algorithm, explainable AI model, facial expression recognition, pattern recognition, segmentation

[Lyons et al., 1998; Lyons, 2021] was not cited in the article. The citation has now been inserted in the section [**4 Results and discussion** Dataset details: jaffedbase facial expression dataset:] and should read: “[The JAFFE database is a foundational, small-scale dataset of posed facial expressions. It consists of 213 grayscale images featuring 10 Japanese female subjects, each displaying the seven prototypical emotions: anger, disgust, fear, happiness, sadness, surprise, and neutral. The JAFFE dataset (Lyons et al., 1998; Lyons, 2021) was used in this study. We thank Prof. Michael Lyons, Miyuki Kamachi, and Jiro Gyoba for providing this valuable resource. For details on the dataset and its proper attribution, please refer to the references below.]”

The following references have been added to the **References** section:

Lyons, M. J., Akamatsu, S., Kamachi, M., and Gyoba, J. (1998). “Coding facial expressions with Gabor wavelets,” in *Proceedings of the Third IEEE International Conference on Automatic Face and Gesture Recognition* (Nara: EEE), 200–205. doi: 10.1109/AFGR.1998.670949

Lyons, M. J. (2021). “Excavating AI” re-excavated: debunking a fallacious account of the JAFFE dataset. *arXiv Preprint arXiv:2107.13998*. doi: 10.48550/arXiv.2107.13998

The original version of this article has been updated.

